# New Non-Bilaterian Transcriptomes Provide Novel Insights into the Evolution of Coral Skeletomes

**DOI:** 10.1093/gbe/evz199

**Published:** 2019-09-13

**Authors:** Nicola Conci, Gert Wörheide, Sergio Vargas

**Affiliations:** 1 Department of Earth and Environmental Sciences, Palaeontology & Geobiology, Ludwig-Maximilians-Universität München, Munich, Germany; 2 GeoBio-Center LMU, Ludwig-Maximilians-Universität München, Munich, Germany; 3 SNSB—Bayerische Staatssammlung für Paläontologie und Geologie, Munich, Germany

**Keywords:** coral calcification, biomineralization, Octocorallia, galaxin, molecular evolution, Scleractinia

## Abstract

A general trend observed in animal skeletomes—the proteins occluded in animal skeletons—is the copresence of taxonomically widespread and lineage-specific proteins that actively regulate the biomineralization process. Among cnidarians, the skeletomes of scleractinian corals have been shown to follow this trend. However, distributions and phylogenetic analyses of biomineralization-related genes are often based on only a few species, with other anthozoan calcifiers such as octocorals (soft corals), not being fully considered. We de novo assembled the transcriptomes of four soft-coral species characterized by different calcification strategies (aragonite skeleton vs. calcitic sclerites) and data-mined published nonbilaterian transcriptome resources to construct a taxonomically comprehensive sequence database to map the distribution of scleractinian and octocoral *skeletome* components. Cnidaria shared no skeletome proteins with Placozoa or Ctenophora, but did share some skeletome proteins with Porifera, such as galaxin-related proteins. Within Scleractinia and Octocorallia, we expanded the distribution for several taxonomically restricted genes such as secreted acidic proteins, scleritin, and carbonic anhydrases, and propose an early, single biomineralization-recruitment event for galaxin sensu stricto. Additionally, we show that the enrichment of acidic residues within skeletogenic proteins did not occur at the Corallimorpharia–Scleractinia transition, but appears to be associated with protein secretion into the organic matrix. Finally, the distribution of octocoral calcification-related proteins appears independent of skeleton mineralogy (i.e., aragonite/calcite) with no differences in the proportion of shared skeletogenic proteins between scleractinians and aragonitic or calcitic octocorals. This points to skeletome homogeneity within but not between groups of calcifying cnidarians, although some proteins such as galaxins and SCRiP-3a could represent instances of commonality.

## Introduction

Cnidaria is a monophyletic lineage of marine and freshwater invertebrates currently comprising ∼9,000 valid species. Their synapomorphy is the cnidocyte, a unique cell type used for locomotion and prey capture (Holstein 1981; Kass-Simon and Scappaticci 2002). Cnidarians have been important reef-building organisms throughout Earth history (Wood 1999) and are the main ecosystem engineers in today’s coral reefs (Wild et al. 2011). Several taxa produce a rigid mineral skeleton made of calcium carbonate (CaCO_3_) and those are found in the anthozoan order Scleractinia and the subclass Octocorallia, as well as in the hydrozoan families of Milleporidae, Stylasteridae, and Hydractiniidae. Calcification apparently has evolved multiple times independently within Cnidaria (i.e., in scleractinians, Romano and Cairns 2000) and hydractinians (Miglietta et al. 2010), and according to molecular clock estimates the origin of the capacity to calcify arose prior to the appearance of cnidarian skeletons in the fossil record (Cartwright and Collins 2007; Erwin et al. 2011; Van et al. 2016).

A common feature of most calcifying organisms is their ability to biologically control and regulate the formation of their skeletons. Although the degree of such control in cnidarians is still debated and the underlying molecular mechanisms are not entirely understood (Tambutté et al. 2011), two main regulatory mechanisms have been described. The first concerns the transport, availability, and concentration of required ions, and involves proteins such as carbonic anhydrases (Jackson et al. 2007; Moya et al. 2008; Bertucci et al. 2011; Le Goff et al. 2016) and bicarbonate transporters (Zoccola et al. 2015), to establish and maintain a chemical (micro)environment that promotes calcium carbonate precipitation (Sevilgen et al. 2019). The second putatively involves the skeletal organic matrix (SOM), an array of proteins (Puverel, Tambutte, Pereira-Mouriès et al. 2005), polysaccharides (Goldberg 2001; Naggi et al. 2018), and lipids (Farre et al. 2010; Reggi et al. 2016) occluded within the mineral fraction of the skeleton (Farre et al. 2010). Skeletal organic matrix proteins (SOMPs) have been suggested to play a role in the promotion or inhibition of crystal growth (Allemand et al. 1998; Clode and Marshall 2003; Puverel et al. 2005), in the regulation of mineral polymorphism (Goffredo et al. 2011) and, more recently, have been shown to regulate the transition from amorphous mineral particles to ordered crystal structures (Von Euw et al. 2017). These proteins are collectively referred to as the “skeletogenic proteins” (Jackson et al. 2007), “biomineralization toolkits” (Drake et al. 2013), or “skeletomes” (Goffredo et al. 2011; Ramos-Silva et al. 2013). The characterization of SOMPs and the study of their evolutionary history is thus essential to unravel the appearance and evolution of biomineralization.

The first protein described and characterized from a coral skeleton was isolated from the organic matrix of the scleractinian coral *Galaxea fascicularis* and thus named galaxin (Fukuda et al. 2003). Galaxins are ubiquitous among scleractinians and putative homologs have been identified in several animal groups, including polychaetes (Sanchez et al. 2007), molluscs (Heath-Heckman et al. 2014), and sea urchins (Sodergren et al. 2006). Although structural similarities with vertebrate usherin (Bhattacharya et al. 2004) led to the proposition of an interaction between galaxin and *type IV* collagen (Bhattacharya et al. 2016), the role of galaxin in cnidarian skeletogenesis remains to be fully resolved (Bhattacharya et al. 2016). Following the first descriptions of single skeletogenic proteins, the advent of tandem mass spectrometry allowed for the simultaneous characterization of several proteins, offering a general overview of coral skeletal proteomes. To date, the proteome of three scleractinian corals: the two acroporids *Acropora digitifera* (Takeuchi et al. 2016) and *Acropora millepora* (Ramos-Silva et al. 2013), and the pocilloporid *Stylophora pistillata* (Drake et al. 2013) have been characterized.

The most abundant fraction of the coral skeletomes so far characterized is represented by acidic proteins (Ramos-Silva et al. 2013; Takeuchi et al. 2016), which supposedly drive crystal nucleation and growth (Wheeler et al. 1981; Addadi et al. 1987). Six acidic proteins have been described from the skeleton of *A. millepora* and two from *S. pistillata*. These include skeletal aspartic acid-rich proteins (SAARPs) (Ramos-Silva et al. 2013) and secreted acidic proteins (SAPs) (Shinzato et al. 2011)—both found in *Acropora* species—and two *S. pistillata* coral acid-rich proteins (CARP4 and CARP5) (Drake et al. 2013). The CARP family (Mass et al. 2013) is of particular interest as recent research has shown how CARPs interact with both aragonite fibers and amorphous calcium carbonate (ACC) during different ontogenetic stages of coral polyps (Akiva et al. 2018). CARPs also appear to be associated with intracellular vesicles putatively transporting Ca^2+^ ions to the extracellular space (Mass et al. 2017).

The nonacidic regions of these acidic proteins match sequences found in other nonbiomineralizing cnidarians and bivalves, making the high occurrence of acidic residues a potential secondary modification linked to biomineralization (Takeuchi et al. 2016).

Surveys of cnidarian transcriptomes and genomes have in fact revealed that only a small proportion of SOMPs in *A. millepora* appears to be taxonomically restricted genes (TRGs) in corals (Ramos-Silva et al. 2013), while the majority of SOMPs (ca. 80% in *A. millepora*) have putative homologs in noncalcifying cnidarians, such as sea anemones and/or *Hydra magnipapillata* (Ramos-Silva et al. 2013). In addition, a recent transcriptome survey of corallimorpharians, skeleton-lacking cnidarians closely related to Scleractinia, has further shown that only six skeletogenic proteins appear to be taxonomically restricted to scleractinian corals (Lin et al. 2017).

So far, however, genomic and transcriptomic surveys have mainly focused on comparisons between scleractinian corals and a limited set of noncalcifying cnidarians (e.g., sea anemones, corallimorpharians, and *Hydra*), systematically overlooking octocorals and calcifying hydrozoans (but see Guzman et al. 2018). Thus, very little information is currently available on the distribution of SOMPs across and within different lineages of calcifying cnidarians and consequently the evolutionary history of their biomineralization-related genes remains largely unexplored.

Here, we conducted an analysis of the distribution of putative coral *biomineralization toolkit* components across Anthozoa. Although functional studies represent the gold standard for the definite identification of genes involved in different biological processes, phylogenetic methods can provide valuable information on the evolution of processes like biomineralization that apparently evolved convergently (Knoll 2003), and help identify candidate proteins for functional studies. Along these lines, our work here allowed us to trace the evolution of skeletogenic protein homologs and investigate observed differences between and within the anthozoan lineages Scleractinia and Octocorallia. In addition, we also compared biomineralization gene repertoires between and within 1) calcifying cnidarians and sponges displaying different calcification strategies (i.e., aragonite vs. calcite deposition, exoskeleton vs. endo-sclerites) such as octocorals and scleractinians or calcareous sponges and the aragonitic demosponge *Vaceletia* sp. and 2) between them and their noncalcifying close relatives. For this, we de novo assembled the transcriptomes of four octocoral species, namely the massive, aragonitic blue coral *Heliopora coerulea*, and calcite producing species *Pinnigorgia flava*, *Sinularia* cf. *cruciata*, and *Tubipora musica*, three sclerites-forming octocorals. These species cover all calcification strategies within Octocorallia. Data-mining of newly generated and publicly available sequence resources was then used to produce fine-scaled phylogenies for selected targets of interest including acidic proteins (e.g., CARPs, SAARPs), galaxin, and carbonic anhydrases. These results contribute to our understanding of the functional diversity and evolutionary history of coral skeletomes.

## Materials and Methods

### Generation of Octocorals Reference Transcriptomes

To obtain reference transcriptomes for our target octocoral species, samples of *H. coerulea*, *T. musica*, *Pinnigorgiaflava*, and *Sinularia* cf. *cruciata*, were mechanically collected from colonies cultured in the aquarium facilities of the Chair for Geobiology & Paleontology of the Department of Earth- and Environmental Sciences at Ludwig-Maximilians-Universität München in Munich (Germany) and kept under control conditions (temperature 25.1 ± 0.5 °C, pH 8.2 ± 0.1) for ca. 1 month before fixation in liquid nitrogen and subsequent storage at −80 °C.

For RNA extraction the samples were homogenized in 1–2 ml TriZol (Thermofisher) using a Polytron PT Homogenizer (Kinematica), and subsequently centrifuged (20 min at 17,000 *g* and 4 °C) to remove remaining skeletal debris. A modified TriZol protocol (Chomczynski and Mackey 1995) was used for RNA purification and the concentration and integrity of the extracted RNA were assessed on a NanoDrop 2100 spectrophotometer and a Bioanalyzer 2100 (Agilent), respectively. For each species, RNA samples with a RIN >8.5 were used to prepare strand-specific libraries that were paired-end sequenced (50 bp reads) on an Illumina HiSeq 2000 sequencer at the EMBL Core Center in Heidelberg (Germany). For *H. coerulea*, additional strand-specific libraries were generated with the SENSE mRNA-Seq Library Prep Kit V2 for Illumina (Lexogen), and sequenced on an Illumina NextSeq 500 at the Kinderklinik und Kinderpoliklinik im Dr von Haunerschen Kinderspital.

Quality control of assembled reads was done with FastQC (www.bioinformatics.babraham.ac.uk) and low-quality reads (*Q* < 28) were removed with the Filter Illumina program from the Agalma-Biolite transcriptome package (Dunn et al. 2013). In addition, reads were mapped against a set of microbial genomes with Bowtie 2 with default parameters (Langmead and Salzberg 2012) and mapping reads were discarded. Transcriptome assembly was performed with Trinity v.2.5.1 (Grabherr et al. 2011). Contigs with a length <300 bp were discarded. Transcriptome completeness was assessed with BUSCO 3.0.2 (Simão et al. 2015) using the Metazoa odb9 data set and protein sequences were predicted with TransDecoder v.3.0.1. Summary statistics for each assembly are provided in table 1. The bioinformatic workflow used is available at https://galaxy.palmuc.org. Reads were deposited at the European Nucleotide Archive (https://www.ebi.ac.uk/ena) under Bioproject number PRJEB30452. Assemblies, untrimmed/trimmed alignments, and output tree files from the various analyses are available at https://gitlab.lrz.de/palmuc/concietal_octoskeletomes.

**Table 1:** Summary Statistics for the Assembled Meta-Transcriptomes

Species	Contigs	N50/Mean Length	BUSCO (C–F–M)
*Heliopora coerulea*	125,310	1,347/967	90.3–7.2–2.5
*Pinnigorgia flava*	84,267	1,125/874	89.4–7.6–3.0
*Sinularia* cf. *cruciate*	69,180	857/721	75.5–18–6.5
*Tubipora musica*	67,632	935/764	86.3–9.7–4.0

Note.—For BUSCO analysis, percentages of complete (C), fragmented (F), and missing (M) orthologs are provided.

### Database Construction and Homologs Search/Analysis

To construct the homolog database (supplementary material 1, Supplementary Material online) of calcification-related proteins, newly assembled transcriptomes were added to a sequence database of representatives of the nonbilaterian metazoan phyla Cnidaria, Porifera, Placozoa, and Ctenophora. To construct the database, publicly available resources for target organisms (excluding tissue-specific transcriptomes) were uploaded on our local Galaxy server (https://galaxy.palmuc.org). Source details for each data set is provided in supplementary material 2, Supplementary Material online. When protein sequences were available, these were directly converted to a protein BLAST database (makeblastdb). Nucleotide sequences were first translated with TransDecoder Galaxy Version 3.0.1 (Haas et al. 2013). For cnidarians, BLAST databases were individually searched (BLASTp, e-value cutoff <1e^−09^) to retrieve putative homologs of coral calcification-related sequences. For the Porifera, Ctenophora, and Placozoa, databases provided in Eitel et al. (2018) were searched using the same criteria listed above. Search queries (supplementary material 3, Supplementary Material online) included scleractinian skeletogenic proteins from *A. millepora* (Ramos-Silva et al. 2013) and *S. pistillata* (Drake et al. 2013), and small cysteine-rich proteins (SCRiPs) from *Orbicella faveolata* (Sunagawa et al. 2009). From *S. pistillata*, two additional SAARP-like acidic proteins that were included in the phylogenetic analysis in Bhattacharya et al. (2016) were additionally used as search queries. Octocoral queries comprised carbonic anhydrases from both *Corallium rubrum* (Debreuil et al. 2012) and *Lobophytum crassum* (Rahman et al. 2006) and scleritin (Debreuil et al. 2012). Features including sequence length and amino acid composition of identified homologs were determined with ProtParam (Gasteiger et al. 2005). To predict the presence of signal peptides, transmembrane regions, and protein domains, SignalP 4.1 (Petersen et al. 2011), TMHMM 2.0 (Sonnhammer et al. 1998), and InteProScan (Jones et al. 2014) were used, respectively.

### Analysis and In Silico Discovery of Acidic Proteins

Amino-acid composition of skeletal acidic proteins and their nonacidic homologs was estimated with ProtParam (https://web.expasy.org/protparam/; last accessed July 20, 2019). The analysis was limited to sequences predicted as complete by TransDecoder (see above). To visually investigate the contribution of changes in acid and basic amino acids to variations in isoelectric point, we performed a principal component analysis (PCA) on sequences grouped according to their phylogeny. Additionally, relative abundance of lysine and aspartic acid for each protein was calculated for the total proteome of seven anthozoan species, for which genomic data are available. Only species with available genomic resources were included in the analysis to avoid potential biases associated with transcriptome assemblies (e.g., missing transcripts due to lack of expression at the time of sampling).

The newly sequenced octocoral transcriptomes were data-mined to investigate the presence of putative biomineralization-related acidic proteins. Assembled contigs from the meta-assemblies were first assigned to either the host or the symbiont using psytrans (https://github.com/sylvainforet/psytrans; last accessed July 17, 2019). Host acidic proteins were identified using a custom script (available in the project repository) using 9% aspartic acid content as the cutoff value and the identified sequences were searched (BLASTp, e-value >e^−05^) against the nonredundant NCBI database. Proteins with no hit or with octocoral-only hits were retained and their distribution mapped across octocoral data sets.

### Homolog Selection for Phylogenetic Analysis

For the phylogenetic reconstruction of acidic proteins, all best-hit sequences identified through the BLASTp searches described above were used. Additionally, nonscleractinian sequences retrieved after BLASTp searches were used as query against scleractinian data sets (using BLASTp, e-value <1e^−09^) (supplementary material 4, Supplementary Material online). If the corresponding scleractinian best-hit differed from those identified using the previous query, sequences were also considered for phylogenetic analysis. The analyses of galaxin sensu stricto (i.e., scleractinian orthologs of *G. fascicularis* galaxin) and galaxin-related proteins (i.e., other putative homologs within and outside scleractinians) are based on all putative homologs (e-value <1e^−09^), with the exception of those matching galaxin-like 1 and 2 (ADI50284.1 and ADI50285.1), as these are exclusively expressed during early stages of calcification (Reyes-Bermudez et al. 2009). Predicted, partial sequences of <200 aa long were excluded. In addition to scleractinians, we surveyed taxa in which galaxin-related proteins have been identified, namely Mollusca, Annelida (Class Polychaeta), and Echinodermata. All resulting sequences were searched, using BLASTp, (e-value <1e^−09^) against the NCBI nonredundant database to avoid including usherin homologs in the data set. Homologous sponge collagen IV sequences were searched using the *type IV* collagen (Q7JMZ8) identified in the homoscleromorph sponge *Pseudocoriticium jarrei* as query. The analysis was limited to the N-terminal NC1 domain. Sequence of each putative homolog was checked for the presence of conserved cysteines (Aouacheria et al. 2006) and added to the collagen IV-spongins data set in Aouacheria et al. (2006). Finally octocoral homologs for the carbonic anhydrases (CA) CruCA1-6 (Le Goff et al. 2016) were searched in all octocoral data sets considered and added to the CAs data set used in Voigt et al. (2014).

### Phylogenetic Analysis

Protein sequences were aligned with MAFFT (Katoh and Standley 2013) and MUSCLE (Edgar 2004) to investigate a possible effect of the aligning algorithm on the final phylogeny. Alignment was followed by a first site selection with Gblocks (Castresana 2002) run within Seaview 4 (Gouy et al. 2010) with the relaxed default parameter, which allows for less stringent site selection. In some instances Gblocks retrieved portions of the signal peptide or did not include well-aligned portions of the sequences. Therefore, a final manual curation step was performed. Untrimmed and trimmed alignments can be found in the project repository and in the untrimmed alignments the excluded/included sites can be visualized in SeaView. Best-fit models were determined with Prottest 3 (Darriba et al. 2011). Maximum-likelihood and Bayesian analyses were performed in PhyML 3.1 (Guindon et al. 2010) from Seaview 4 (Gouy et al. 2010) with 500 bootstrap replicates, and MrBayes 3.2 (nruns = 2, samplefreq = 100; Huelsenbeck and Ronquist 2001; Ronquist et al. 2012), respectively. Effective Sample Sizes (EES > 200) and burn-in fractions (0.20–0.25) were determined with Tracer v.1.6 (http://tree.bio.ed.ac.uk/software/tracer/).

## Results

### Distribution Analysis of Skeletogenic Proteins

**Fig. 1.**
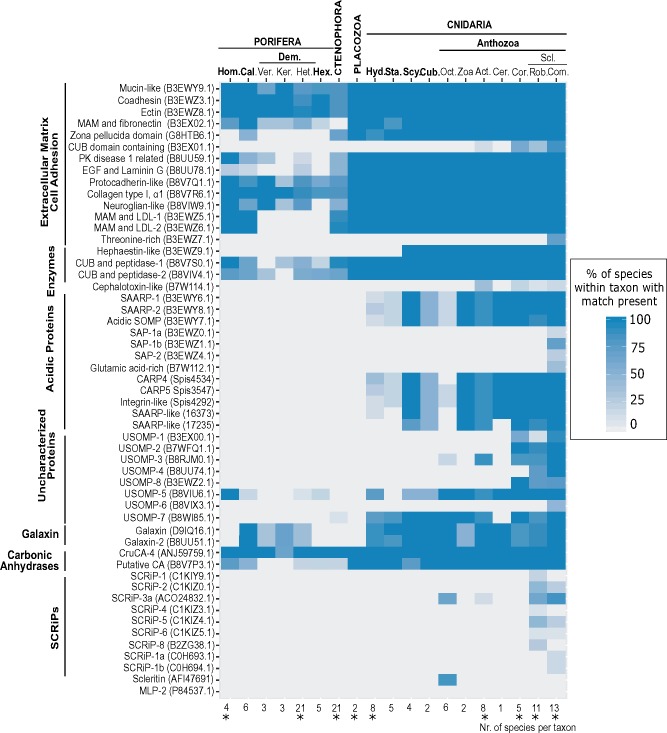
—Pattern of presence of homologs (BLASTp, e-value <1e^−09^) of coral biomineralization-related protein across early branching metazoans. Lower *x* axis indicates number of species surveyed within a particular group. Asterisk: genomic data available for at least one species within the group. Protein categories “Extracellular matrix—Cell Adhesion,” “Enzymes,” “Uncharacterized Proteins,” and “Galaxins” based on Ramos-Silva et al. (2013). Taxa in capital and bold, phyla; taxa in bold, classes; normal text: subclasses or lower taxonomic levels; Hom, Homoscleromorpha; Cal, Calcarea; Ver, Verongimorpha; Ker, Keratosa; Het, Heteroscleromorpha; Dem, Demospongiae; Hex, Hexactinellida; Hyd, Hydrozoa; Sta, Staurozoa; Scy, Scyphozoa; Cub, Cubozoa; Oct, Octocorallia; Zoa, Zoantharia; Act, Actiniaria; Cer, Ceriantharia; Cor, Corallimorpharia; Scl, Scleractinia; Rob, Robusta (Scleractinia); Com, Complexa (Scleractinia).

The distribution analysis of SOMP homologs resulted in diverse presence/absent patterns (fig. 1). Carbonic anhydrases, peptidases, and extracellular/adhesion proteins display the widest taxonomic distribution, although similarity was often limited to conserved domains within protein sequences. In Porifera and Cnidaria however, differences could also be observed in terms of domain presence. Among sponges, the zona pellucida (ZP) domain was observed only in Calcarea, while the MAM domain appears to be absent in Demospongiae, as reported in Riesgo et al. (2014). In Cnidaria the cupredoxin domain could not be retrieved in Hydrozoa. In contrast, all SAPs and all small cysteine-rich (SCRiPs) proteins with the sole exception of SCRiP-3a (ACO24832.1), which was detected in Scleractinia and Octocorallia, showed the most taxonomically restricted distribution. Despite the presence of proteins found only among certain scleractinian families (e.g., SAPs, Threonine-rich protein), no protein hitherto isolated from the skeleton of *A. millepora* was found here restricted to acroporids. No protein was exclusively found in “Cnidaria+Placozoa” or “Cnidaria+Ctenophora,” while a small set of coral SOMPs appeared to possess homologs in Cnidaria and Porifera. These include galaxin-related proteins and the uncharacterized *A. millepora* protein USOMP-5 (B8VIU6.1). Although absent in Homoscleromorpha and Hexactinellida, galaxin-related proteins are ubiquitous among calcareous sponges and also found in all three currently described subclasses of Demospongiae. Within Heteroscleromorpha however, differences were observed between groups as no galaxin-related protein was retrieved from the genome of *Amphimedon queenslandica* (Srivastava et al. 2010), while a significant hit was returned from the genome of *Tethya wilhelma* (Francis et al. 2017). The highest occurrence rate for USOMP-5 homologs in sponges was observed in Homoscleromorpha, but matches were detected in all groups. Although no domain was originally reported for B8VIU6.1 in *A. millepora* (Ramos-Silva et al. 2013), analysis of matching sequences from sponges revealed the presence of fibrinogen-related subdomains (IPR014716, IPR036056) within the protein (supplementary fig. 1, Supplementary Material online). Domain location partly overlaps the conserved region of the protein, and might thus explain the detected local similarity.

Cnidaria exclusive proteins showed diverse presence/absence patterns with some SOMPs retrieving putative homologous sequences across the phylum’s classes while others could be only found restricted to few anthozoan families. Acidic proteins SAARPs and CARPs produced significant BLASTp matches among several cnidarian groups, although the presence of acidic regions (i.e., sequences segments enriched in aspartic and glutamic acid) appears to be characteristic of scleractinian corals (see below). Within Octocorallia, homologs of SAARPs and CARPs could be retrieved only in the precious coral *C. rubrum* using BLAST. Nonetheless, in silico analyses of octocoral transcriptomes identified octocoral exclusive, secreted, aspartic-rich proteins in different species. These sequences did not produce significant BLASTp hits against public databases. Members of the SAP acidic family were, on the other hand, detected solely in complex scleractinians, but not only in acroporids as previously suggested (Shinzato et al. 2011; Takeuchi et al. 2016). Homologs of SAP-1b (B3EWZ1.1) are in fact also present within families Dendrophylliidae and Agariciidae. Other uncharacterized proteins (USOMPs) displayed varying presence/absence patterns. USOMP-7 (B8WI85.1) and USOMP-3 (B8RJM0.1) were found across Cnidaria and Anthozoa, respectively. The latter also represents the only difference we detected between aragonitic and calcitic octocorals as this protein was solely found in *H. coerulea*. As reported in Lin et al. (2017), USOMP-1 is present in anemones and scleractinians, while both USOMP-2 and USOMP-8 first appear in corallimorphs. Finally, USOMP-4 and USOMP-6 are restricted to scleractinians, although the first is shared by complex and robust corals and the second was only found in the families Acroporidae and Agariciidae.

No significant match was detected among octocorals for the acidic carbonic anhydrase MLP-2 (Rahman et al. 2006), while we retrieved homologs across the group for both scleritin and five (CruCA1-5) of the six carbonic anhydrases described for *C. rubrum* (supplementary figs. 8 and 9, Supplementary Material online) (Le Goff et al. 2016), including the putative skeletogenic CruCA-4. No difference has thus been observed here for octocoral calcification-related proteins between aragonite and calcite-deposing species.

### Phylogenetic Analysis of Acidic Proteins

**Fig. 2.**
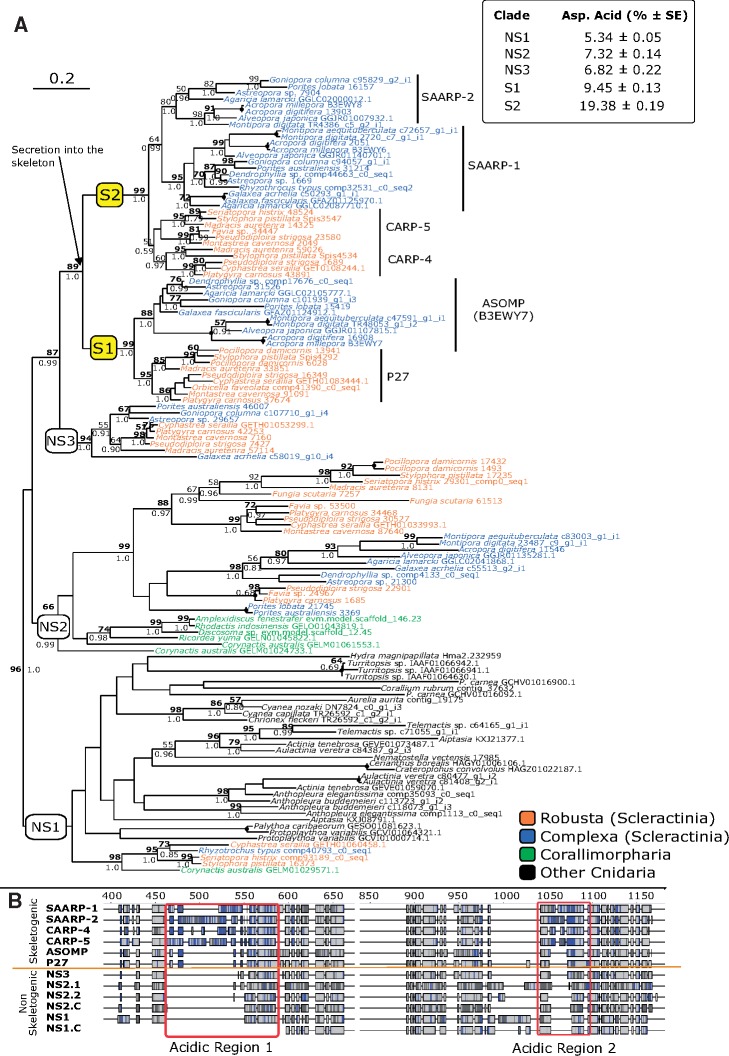
—(*a*) Phylogenetic tree (ML, 500 bootstrap replicates) of scleractinian acidic proteins and putative homologs in other cnidarian groups. Best-fit model: WAG+F + G+I. Tree displayed in figure based on protein sequences aligned with MAFFT. MUSCLE alignment and tree available in supplementary figure 2, Supplementary Material online. Bold number: node supported (>50) also in MUSCLE phylogeny. Dot on node indicates full support (100% bootstrap, 1.0 posterior probability) in both phylogenies. Support for nodes with bootstrap <50 not shown regardless of posterior probability value. Skeletogenic clades (S) (highlighted in yellow) include acidic proteins found in coral skeletons (Drake et al. 2013; Ramos-Silva et al. 2013). NS (nonskeletogenic) clades: acidic proteins not extracted from coral skeletons. (*b*) Consensus sequences (60%) alignment for each clade. Alignment shows the position and distribution of acidic residues (aspartic and glutamic acid) highlighted in blue. Light gray: other conserved residues. Dark gray: nonconserved residues. Complete alignment available in the project repository. When corallimorph sequences were present in a clade, these were analyzed separately to highlight difference with scleractinian proteins Corallimorph consensus sequences IDs end in “.C”. NS2 clade was split into NS2.1 (includes *Porites australiensis* 3369, *Porites lobata* 21745, *Favia* sp. 24967, *Platygyra carnosus* 1685 and *Pseudodiploria strigosa* 22901) and NS2.2 (all other scleractinian sequences) because the position of NS2.1 was not congruent between phylogenies and was also retrieved as sister group to the rest of NS2 scleractinian proteins (supplementary fig. 2, Supplementary Material online). Top right corner: mean (± SE) content (%) of aspartic acid within acidic proteins. Average estimated on predicted complete sequences only.

Phylogenetic analysis split acidic proteins and their nonacidic homologs into five well-supported clades: two of these (marked as “S” for skeletogenic clades) are occupied by proteins found occluded in coral skeletons. Only scleractinians are represented within these groups. S1 contains homologs for the acidic SOMP (B3EWY7) and P27 isolated from *A. millepora* (Ramos-Silva et al. 2013) and *S. pistillata* (Drake et al. 2013), respectively. Both of these proteins display shorter acidic regions and a lower aspartic acid content compared with SAARPs and CARPs, which occupy clade S2. Tree topology within this group did however change between phylogenies obtained using different alignment methods (i.e., MUSCLE vs. MAFFT). In the MAFFT-based phylogeny displayed below, (fig. 2*a*) CARPs and SAARPs are split into two distinct subgroups although bootstrap support was low. All other sequences were divided among three nonskeletogenic (NS) clades. Taxonomic diversity for these groups differed and ranged from Cnidaria (NS1) to scleractinians (NS3), while NS2 contained scleractinians and corallimorphs.

When aligned with MUSCLE, SAARP-2 grouped with both CARPs but support was again low ( supplementary fig. 2, Supplementary Material online). The internal topology of clade NS2 was also affected. When aligned with MUSCLE both *Porites* sequences, together with *Favia* sp. 24967, *Platygyra carnosus* 1685, and *Pseudodiploria strigosa* 22901, were placed as sister group to other scleractinians (supplementary fig. 2, Supplementary Material online). The split between corallimorphs and scleractinians within NS2 was nevertheless retrieved in both phylogenies. All other cnidarian sequences grouped with the scleractinian homologs of *S. pistillata* protein 17235 (NS1). As previously reported (Takeuchi et al. 2016), similarity between acidic proteins and their putative homologs is restricted to nonacidic regions. Analysis of clade-consensus sequences shows that the appearance of the aspartic acid-rich regions corresponds with the secretion of the proteins into the skeleton matrix and not with the shift between corallimorphs and scleractinian sequences (fig. 2*b*). Within B3EWY7-P27 the increment in aspartic acid appears restricted to the first acidic region, and it then continues in SAARP1 and CARP4, ultimately escalating in SAARP-2 and CARP-5 which exhibit the longest extension of the first acidic region. The transition from nonskeletogenic to skeletogenic proteins is also marked by a sharp decrease in protein isoelectric point that is mainly driven by the increase in aspartic acid (see above) and a concurrent decline in lysine content (supplementary material 2 and fig. 10, Supplementary Material online). These trends do not apply to the whole scleractinian proteome but are specific to skeletal proteins. Finally, the amount of glutamic acid does appear to remain unaltered between the NC and S clades, although principal components analysis based on sequence composition points to lower contents in clade S2 compared with clade S1.

### Galaxin and *Type IV* Collagen

**Fig. 3.**
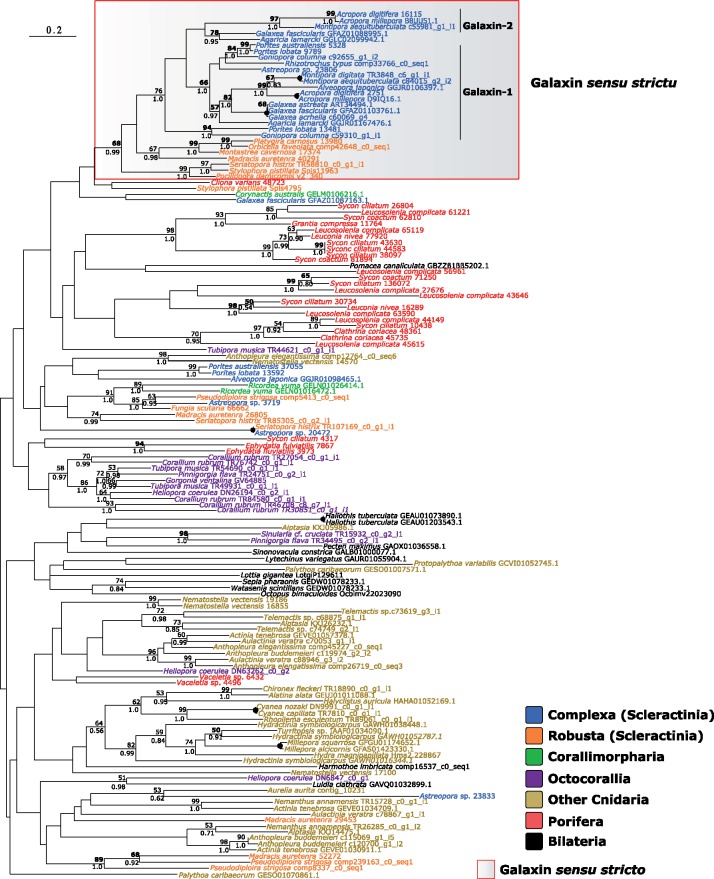
—Phylogenetic analysis (ML; 500 bootstrap replicates) of metazoan galaxin-related proteins. Tree displayed in figure based on protein sequences aligned with MAFFT. MUSCLE alignment and tree available in supplementary material 3, Supplementary Material online. MUSCLE-based phylogeny in supplementary figure 3, Supplementary Material online. Bold number: node supported (>50) also in MUSCLE phylogeny. Dot on node indicates full support (100 bootstrap, 1.0 posterior probability) in both phylogenies. Support for nodes with bootstrap <50 not shown regardless of posterior probability value.

Phylogenetic analysis of metazoan galaxin-related proteins revealed high degrees of polyphyly among lineages both at the phylum and lower levels, with only terminal nodes displaying moderate to high support (fig. 3). Taxonomically uniform clades were observed in both MAFFT- and MUSCLE-based phylogenies. These included galaxin-related proteins from calcareous sponges, octocorals and Hydrozoa. However, for the vast majority of these clades, both support and topology were influenced by the alignment algorithm employed.

The exception to this general pattern is a scleractinian-only clade comprising both complex and robust corals. The group includes both *A. millepora* skeletogenic (D9IQ16.1 and B8UU51.1) and the original *G. fascicularis* galaxins. The unifying feature of this clade is the RXRR endoprotease target motif described in Fukuda et al. (2003) (supplementary fig. 4, Supplementary Material online). This RXRR motif is not unique to scleractinians, but it was not detected in any other galaxin-related protein within the group. Its presence thus appears to effectively discriminate a group of galaxins, here dubbed galaxins sensu stricto, from galaxin-related proteins.

**Fig. 4.**
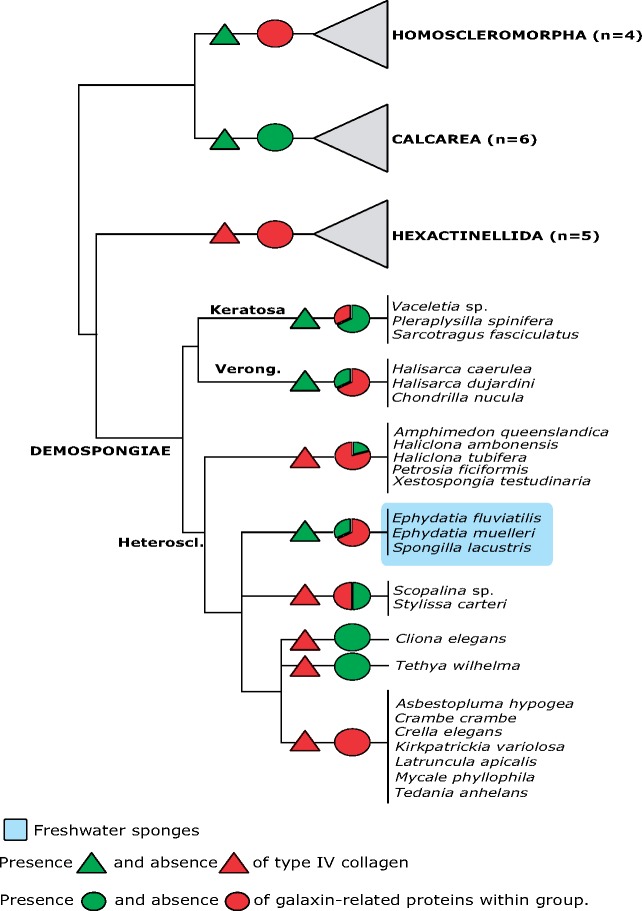
—Presence-absence analysis of *type IV* collagen and galaxin-related proteins within Porifera. For galaxin-related proteins, data are presented as percentage of species within group in which one significant match (BLASTp, e-value <1e^−10^) was detected. When present, collagen IV was found in all species considered for a particular taxon (supplementary material 1, Supplementary Material online). Phylogenetic relationships between sponge classes based on Simion et al. (2017). Phylogeny of Demospongiae based on Morrow and Cárdenas (2015). Heteroscl, Heteroscleromorpha; Verong, Verongimorpha.

Although the monophyly of galaxins sensu stricto was robust to the alignment algorithm, its internal topology was affected, with galaxin-2s and *Rhizotrochus typus* sequences nesting either within Complexa (MAFFT) or Robusta (MUSCLE). When performing the analysis on galaxin sensu stricto sequences only, galaxin-2 sequences concordantly grouped together with other complex scleractinians (supplementary fig. 5, Supplementary Material online), in agreement with the topology derived from the MAFFT alignment and presented in figure 3. To investigate putative interactions between galaxin-related proteins and collagen IV, we mapped the distribution of both proteins in Porifera, as both are present but not ubiquitous in the phylum (fig. 4).

As for galaxin-related proteins, *type IV* collagen is present across calcareous sponges, while Homoscleromorpha are the only sponge class with collagen IV but no galaxin homologs. Collagen IV is also present in both keratose and verongimorph sponges, while within Heteroscleromorpha it appears associated with the freshwater environment. Finally, neither protein is present in glass sponges (Hexactinellida).

Both phylogenetic analysis resulted in monophyly of collagen IV for all three sponge classes in which the protein is present (supplementary figs. 6 and 7, Supplementary Material online). In one instance (MAFFT-based phylogeny), support for monophyly of Porifera was also retrieved.

## Discussion

A common feature of skeletal proteomes is the presence of both taxonomically widespread proteins with homologs in other, not necessarily calcifying, organisms and of lineage-specific innovations or TRGs (Ramos-Silva et al. 2013; Kocot et al. 2016). The diversity of evolutionary histories characterizing skeletogenic proteins, make phylogenetic analyses and gene distribution maps a necessary step to examine the evolution of biomineralization. Key to this is the need for extensive taxon sampling. Here, we data-mined available resources across nonbilaterian metazoans to examine the distribution of skeletogenic proteins, allowing comparative investigations of the genetic repertoires of diverse calcifying organisms, and produced detailed phylogenies for key components of coral biomineralization toolkits. As most of the information presented is based on transcriptomic data, the distribution patterns observed for the studied homologs may be underestimated due to transcriptome incompleteness. To mitigate possible sampling biases, and to reduce the impact of different sampling sizes across taxa, gene presence within a taxon was presented and discussed as relative abundance. Secondly, for several SOMPs, evidence of indirect involvement in calcification is still lacking and a random incorporation in the skeleton cannot be excluded (Takeuchi et al. 2016). We therefore focus our discussion on proteins—that is, acidic SOMPs and galaxin—for which proteomic-independent evidence for a role in skeleton formation is available (Reyes-Bermudez et al. 2009; Mass et al. 2017; Von Euw et al. 2017).

Distribution analysis reflected evolutionary heterogeneity, with homologs being present across phyla or restricted to selected families. Although a few coral skeletogenic proteins remain largely restricted taxonomically, increased taxon sampling resulted in the expansion of their taxonomic distribution. In these cases, the most common pattern was their presence across phyla, or limited to Cnidaria or Scleractinia, which does not support the involvement of these proteins in biomineralization across groups. SCRiP-3a and galaxin-related proteins are, however, potential targets for future (functional) research, because of their presence pattern (e.g., SCRiP-3a found among calcifying anthozoans only). The distribution of the latter within sponges is of particular interest as we show that these proteins are present in all calcifying species, regardless of their taxonomic position. Moreover, the presence of multiple potential galaxin homologs among calcifying Calcarea and their absence among homoscleromorphs and glass sponges, supports their potential involvement in calcium carbonate biomineralization.

As for galaxin-related proteins, collagen IV appears either ubiquitous or absent in different sponge classes, while a patchy distribution can be observed among groups of Demospongiae. Within Heteroscleromorpha presence of *type IV* collagen appears however, as previously hypothesized by Riesgo et al. (2014), associated with the freshwater environment, but among keratose sponges it could be related to the collagenous framework of their organic skeletons (Junqua et al. 1974; Germer et al. 2015).

Scleractinian TRGs also exhibited a wider variety of distribution patterns, ranging from being present across both robust and complex corals down to small set of scleractinian families only (e.g., galaxin-2 and SAPs). The former are of particular interest for the evolution of corals. Although different time estimates have been put forward, the accepted consensus places the divergence of Complexa and Robusta in the Palaeozoic, prior to the (ca. 240 Ma) appearance of fossil modern scleractinians in the early/mid-Triassic (Romano and Palumbi 1996; Stolarski et al. 2011; Chuang et al. 2017). The discovery of palaeozoic scleractinian-like fossils does support a Palaeozoic origin for the group, with consequent fossil gaps likely being caused by poor preservation or abiotic conditions hindering the deposition of skeletons (Stolarski et al. 2011). Whether a particular skeletogenic protein was available to the common ancestor of complex and robust scleractinian corals is thus of particular evolutionary interest as it allows to determine which components of the biomineralization toolkit preceded the Triassic appearance of the skeleton and whether putative palaeozoic scleractinians had access to the same molecular machinery currently employed by modern representatives of the group. In this regard, one biomineralization-related event that might have preceded the Complexa-Robusta divergence appears to be the expansion in the number of acidic residues within acidic proteins. The close phylogenetic relationship between P27 (*S. pistillata*) and B3EWY7 (*A. millepora*)—which are best BLAST reciprocal hits—is supported by the high similarity in the location and structure of their acidic regions. Moreover, such increases in aspartic acid could not be observed within scleractinian total proteomes. This excludes the possibility of higher aspartic content representing a lineage-specific innovation, and supports it being a biomineralization-related event.

A similar scenario could also apply to galaxin sensu stricto. These proteins have been proposed to have been independently recruited by and within scleractinians families (e.g., Pocilloporidae, Bhattacharya et al. 2016), implying that the protein acquired its calcification-related role after the Complexa-Robusta split. However, the presence of representatives of both robust and complex corals within the galaxin sensu stricto clade described here points to an alternative scenario in which the recruitment of galaxin for biomineralization occurred only once, prior to the divergence of these clades. On the other hand, the relationship between *A. millepora* galaxin 1 and galaxin 2 remains uncertain due to the current lack of support in phylogenetic analyses. Despite this, phylogenetic analysis allows to confidently argue that the protein is present in the family Agariciidae and Acroporidae and it should be considered a true (sensu stricto) galaxin. One aspect that remains unsolved concerns the evolutionary history of galaxin sensu stricto outside Scleractinia. Extensive divergence between scleractinians and other cnidarians could have eroded the evolutionary signal in these proteins (Forêt et al. 2010). Nevertheless, inability to obtain supported phylogenies for galaxin proteins might also be currently exacerbated by the inclusion of several, possibly functionally diverse, galaxin-related proteins in phylogenetic analyses. Similarity between galaxin sensu stricto and other galaxin-related proteins is often low and restricted to di-cysteine motifs (personal observations). Combined with the current lack of additional defining features for galaxins, this complicates BLAST-based homolog selection which can lead to the inclusion of unrelated proteins within protein data sets in phylogenetic analyses. Although our analysis is not immune to these limitations, expanding homolog selection beyond best-matches only helped to identify putative erroneous inclusions. An example described here is the *Fungia scutaria* protein 6662. When a galaxin sensu stricto sequence is used as a query, this sequence is the only hit in *F. scutaria*. Including multiple galaxin BLAST matches per species did reveal however that the protein is instead a scleractinian galaxin-related protein. The presence of “undetected” galaxin-related proteins, erroneously considered genuine galaxin sensu stricto homologs, could thus explain the previously described galaxin polyphyly (Bhattacharya et al. 2016).

Finally, in contrast to scleractinians, octocoral TRGs were found conserved across soft-coral taxa showing similar distributions. Although the number of calcification-related genes in soft corals is currently extremely limited, intra-Octocorallia analyses are of potential interest, as they might allow for the identification of differences between calcite and aragonite-deposing species, and similarities between aragonitic animals within Anthozoa (i.e., *H. coerulea* and Scleractinia). The presence of TRGs (such as scleritin) in species belonging to all the three major octocoral clades (McFadden et al. 2006), indicates that TRGs, although restricted to octocorals, were present in the common ancestor of the subclass. On one hand, this points toward a certain degree of commonality in spite of the different biomineralization strategies (calcite vs. aragonite). On the other hand, it could be related to scenarios in which, as galaxin sensu stricto (Forêt et al. 2010), the protein played a different ancestral function with subsequent lineage-specific recruitment events for biomineralization.

Here, we conducted a distribution and phylogenetic analysis of coral biomineralization genes to provide a comprehensive homolog mapping and fine-scaled phylogenies of selected genes. Through a relatively broad taxon sampling, our work allowed us to detect similarities and differences between different taxonomic groups and investigate patterns of protein presence/absence associated with skeleton polymorph. This led to the postulation of a single recruitment for calcification of galaxin sensu stricto and provided a detailed phylogeny of coral acidic proteins that revealed the increase of acidic residues during cnidarian evolution. We also provide insights into the evolution of proteins likely involved in biomineralization, such as sponge collagen IV. With the inclusion of four new octocoral transcriptomes, we have closed the existing taxon bias toward certain cnidarian taxa, specifically scleractinian corals, however gaps still exist. For instance, groups like calcifying hydrozoans remain unexplored and their inclusion in future studies on biomineralization will certainly contribute to our understanding of this process in Cnidaria. Proteomic investigations of the SOM of calcifying cnidarians other than scleractinian corals and of sponges might reveal the presence of shared skeletome components adding support to the transcriptomic presence patterns described here, and will help discover lineage-specific innovations linked to calcification in these groups. 

## Supplementary Material

Supplementary data are available at *Genome Biology and Evolution* online.

## Supplementary Material

evz199_Supplementary_Data

## References

[evz199-B1] Addadi L, Moradian J, Shay E, Maroudas NG, Weiner S. 1987 A chemical model for the cooperation of sulfates and carboxylates in calcite crystal nucleation: relevance to biomineralization. Proc Natl Acad Sci U S A. 84(9):2732–2736. 16593827 10.1073/pnas.84.9.2732 PMC304732

[evz199-B2] Akiva A, et al 2018 Minerals in the pre-settled coral *Stylophora pistillata* crystallize via protein and ion changes. Nat Commun. 9(1):1880. 29760444 10.1038/s41467-018-04285-7 PMC5951882

[evz199-B3] Allemand D, TambuttE E, Girard JP, Jaubert J. 1998 Organic matrix synthesis in the scleractinian coral *Stylophora pistillata*: role in biomineralization and potential target of the organotin tributyltin. J Exp Biol. 201(Pt 13):2001–2009. 9622572 10.1242/jeb.201.13.2001

[evz199-B4] Aouacheria A, et al 2006 Insights into early extracellular matrix evolution: spongin short chain collagen-related proteins are homologous to basement membrane type IV collagens and form a novel family widely distributed in invertebrates. Mol Biol Evol. 23(12):2288–2302. 16945979 10.1093/molbev/msl100

[evz199-B5] Bertucci A, Tambutté S, Supuran CT, Allemand D, Zoccola D. 2011 A new coral carbonic anhydrase in *Stylophora pistillata*. Mar Biotechnol. 13(5):992–1002. 21318259 10.1007/s10126-011-9363-x

[evz199-B6] Bhattacharya D, et al 2016 Comparative genomics explains the evolutionary success of reef-forming corals. Elife. 5:e13288. 10.7554/eLife.13288 PMC4878875 27218454

[evz199-B7] Bhattacharya G, Kalluri R, Orten DJ, Kimberling WJ, Cosgrove D. 2004 A domain-specific usherin/collagen IV interaction may be required for stable integration into the basement membrane superstructure. J Cell Sci. 117(Pt 2):233–242. 14676276 10.1242/jcs.00850

[evz199-B8] Cartwright P, Collins A. 2007 Fossils and phylogenies: integrating multiple lines of evidence to investigate the origin of early major metazoan lineages. Integr Comp Biol. 47(5):744–751. 21669755 10.1093/icb/icm071

[evz199-B9] Castresana J. 2000. Selection of conserved blocks from multiple alignments for their use in phylogenetic analysis. Mol Biol Evol 17:540–552. 10.1093/oxfordjournals.molbev.a026334 10742046

[evz199-B10] Chomczynski P, Mackey K. 1995 Short technical reports. Modification of the TRI reagent procedure for isolation of RNA from polysaccharide- and proteoglycan-rich sources. Biotechniques 19(6):942–945. 8747660

[evz199-B11] Chuang Y, et al 2017 Loss and gain of group I introns in the mitochondrial Cox1 gene of the Scleractinia (Cnidaria; Anthozoa.). Zool Stud. 56. 10.6620/ZS.2017.56-09 PMC6517707 31966208

[evz199-B12] Clode PL, Marshall AT. 2003 Calcium associated with a fibrillar organic matrix in the scleractinian coral *Galaxea fascicularis*. Protoplasma 220(3–4):153–161. 12664279 10.1007/s00709-002-0046-3

[evz199-B13] Darriba D, Taboada GL, Doallo R, Posada D. 2011 ProtTest 3: fast selection of best-fit models of protein evolution. Bioinformatics 27(8):1164–1165. 21335321 10.1093/bioinformatics/btr088 PMC5215816

[evz199-B14] Debreuil J, et al 2012 Molecular cloning and characterization of first organic matrix protein from sclerites of red coral, *Corallium rubrum*. J Biol Chem. 287(23):19367–19376. 22505718 10.1074/jbc.M112.352005 PMC3365975

[evz199-B15] Drake JL, et al 2013 Proteomic analysis of skeletal organic matrix from the stony coral *Stylophora pistillata*. Proc Natl Acad Sci U S A. 110(10):3788–3793. 23431140 10.1073/pnas.1301419110 PMC3593878

[evz199-B16] Dunn CW, Howison M, Zapata F. 2013 Agalma: an automated phylogenomics workflow. BMC Bioinformatics 14:330. 24252138 10.1186/1471-2105-14-330 PMC3840672

[evz199-B17] Edgar RC. 2004 MUSCLE: multiple sequence alignment with high accuracy and high throughput. Nucleic Acids Res. 32(5):1792–1797. 15034147 10.1093/nar/gkh340 PMC390337

[evz199-B18] Eitel M, et al 2018 Comparative genomics and the nature of placozoan species. PLoS Biol. 16(7):e2005359. 30063702 10.1371/journal.pbio.2005359 PMC6067683

[evz199-B19] Erwin DH, et al 2011 The Cambrian conundrum: early divergence and later ecological success in the early history of animals. Science 334(6059):1091–1097. 22116879 10.1126/science.1206375

[evz199-B20] Farre B, Cuif J-P, Dauphin Y. 2010 Occurrence and diversity of lipids in modern coral skeletons. Zoology (Jena) 113(4):250–257. 20800460 10.1016/j.zool.2009.11.004

[evz199-B21] Forêt S, et al 2010 New tricks with old genes: the genetic bases of novel cnidarian traits. Trends Genet. 26(4):154–158. 20129693 10.1016/j.tig.2010.01.003

[evz199-B22] Francis WR, et al 2017 The genome of the contractile demosponge *Tethya wilhelma* and the evolution of metazoan neural signalling pathways. BioRxiv 120998; doi: https://doi.org/10.1101/120998.

[evz199-B23] Fukuda I, et al 2003 Molecular cloning of a cDNA encoding a soluble protein in the coral exoskeleton. Biochem Biophys Res Commun. 304(1):11–17. 12705876 10.1016/s0006-291x(03)00527-8

[evz199-B24] Gasteiger E, et al 2005 Protein identification and analysis tools on the ExPASy server In: Walker JM, editor. The proteomics protocols handbook. Totowa (NJ): Humana Press p. 571–607.

[evz199-B25] Germer J, Mann K, Wörheide G, Jackson DJ. 2015 The skeleton forming proteome of an early branching metazoan: a molecular survey of the biomineralization components employed by the coralline sponge *Vaceletia* sp. PLoS One 10(11):e0140100. 26536128 10.1371/journal.pone.0140100 PMC4633127

[evz199-B26] Goffredo S, et al 2011 The skeletal organic matrix from Mediterranean coral *Balanophyllia europaea* influences calcium carbonate precipitation. PLoS One 6(7):e22338. 21799830 10.1371/journal.pone.0022338 PMC3142144

[evz199-B27] Goldberg WM. 2001 Acid polysaccharides in the skeletal matrix and calicoblastic epithelium of the stony coral *Mycetophyllia reesi*. Tissue Cell. 33(4):376–387. 11521954 10.1054/tice.2001.0191

[evz199-B28] Gouy M, Guindon S, Gascuel O. 2010 SeaView version 4: a multiplatform graphical user interface for sequence alignment and phylogenetic tree building. Mol Biol Evol. 27(2):221–224. 19854763 10.1093/molbev/msp259

[evz199-B29] Grabherr MG, et al 2011 Full-length transcriptome assembly from RNA-Seq data without a reference genome. Nat Biotechnol. 29(7):644–652. 21572440 10.1038/nbt.1883 PMC3571712

[evz199-B30] Guindon S, et al 2010 New algorithms and methods to estimate maximum-likelihood phylogenies: assessing the performance of PhyML 3.0. Syst Biol. 59(3):307–321. 20525638 10.1093/sysbio/syq010

[evz199-B82] Guzman C, Shinzato C, Lu TM, Conaco C. 2018. Transcriptome analysis of the reef-building octocoral, *Heliopora coerulea*. Sci. Rep 8: 8397. 10.1038/s41598-018-26718-5 PMC5976621 29849113

[evz199-B31] Haas BJ, et al 2013 De novo transcript sequence reconstruction from RNA-seq using the Trinity platform for reference generation and analysis. Nat Protoc. 8(8):1494–1512. 23845962 10.1038/nprot.2013.084 PMC3875132

[evz199-B32] Heath-Heckman EAC, et al 2014 Shaping the microenvironment: evidence for the influence of a host galaxin on symbiont acquisition and maintenance in the squid-*Vibrio* symbiosis. Environ Microbiol. 16(12):3669–3682. 24802887 10.1111/1462-2920.12496 PMC4224630

[evz199-B33] Holstein T. 1981 The morphogenesis of nematocytes in *Hydra* and *Forsklia*: an ultrastructural study. J Ultrastruct Res. 75(3):276–290. 7277568 10.1016/s0022-5320(81)80085-8

[evz199-B34] Huelsenbeck JP, Ronquist F. 2001 MRBAYES: Bayesian inference of phylogenetic trees. Bioinformatics 17(8):754–755. 11524383 10.1093/bioinformatics/17.8.754

[evz199-B35] Jackson DJ, Macis L, Reitner J, Degnan BM, Wörheide G. 2007 Sponge paleogenomics reveals an ancient role for carbonic anhydrase in skeletogenesis. Science 316(5833):1893–1895. 17540861 10.1126/science.1141560

[evz199-B36] Jones P, et al 2014 InterProScan 5: genome-scale protein function classification. Bioinformatics 30(9):1236–1240. 24451626 10.1093/bioinformatics/btu031 PMC3998142

[evz199-B37] Junqua S, Robert L, Garrone R, Pavans de Ceccatty M, Vacelet J. 1974 Biochemical and morphological studies on collagens of horny sponges. Ircinia filaments compared to spongines. Connect Tissue Res. 2(3):193–203. 4373212 10.3109/03008207409152244

[evz199-B38] Kass-Simon G, Scappaticci AA Jr. 2002 The behavioral and developmental physiology of nematocysts. Can J Zool. 80(10):1772–1794.

[evz199-B39] Katoh K, Standley DM. 2013 MAFFT multiple sequence alignment software version 7: improvements in performance and usability. Mol Biol Evol. 30(4):772–780. 23329690 10.1093/molbev/mst010 PMC3603318

[evz199-B40] Knoll AH. 2003 Biomineralization and evolutionary history. Rev Min Geochem. 54(1):329–356.

[evz199-B41] Kocot KM, Aguilera F, McDougall C, Jackson DJ, Degnan BM. 2016 Sea shell diversity and rapidly evolving secretomes: insights into the evolution of biomineralization. Front Zool. 13:23. 27279892 10.1186/s12983-016-0155-z PMC4897951

[evz199-B42] Langmead B, Salzberg SL. 2012 Fast gapped-read alignment with Bowtie 2. Nat Methods. 9(4):357–359. 22388286 10.1038/nmeth.1923 PMC3322381

[evz199-B43] Le Goff C, et al 2016 Carbonic anhydrases in Cnidarians: novel perspectives from the Octocorallian *Corallium rubrum*. PLoS One 11(8):e0160368. 27513959 10.1371/journal.pone.0160368 PMC4981384

[evz199-B44] Lin M-F, et al 2017 Analyses of Corallimorpharian transcriptomes provide new perspectives on the evolution of calcification in the Scleractinia (corals). Genome Biol Evol. 9(1):150–160. 28158437 10.1093/gbe/evw297 PMC5604590

[evz199-B45] Mass T, Drake JL, Heddleston JM, Falkowski PG. 2017 Nanoscale visualization of biomineral formation in coral proto-Polyps. Curr Biol. 27(20):3191–3196.e3. 29033329 10.1016/j.cub.2017.09.012

[evz199-B46] Mass T, et al 2013 Cloning and characterization of four novel coral acid-rich proteins that precipitate carbonates in vitro. Curr Biol. 23(12):1126–1131. 23746634 10.1016/j.cub.2013.05.007

[evz199-B47] McFadden CS, France SC, Sánchez JA, Alderslade P. 2006 A molecular phylogenetic analysis of the Octocorallia (Cnidaria: anthozoa) based on mitochondrial protein-coding sequences. Mol Phylogenet Evol. 41(3):513–527. 16876445 10.1016/j.ympev.2006.06.010

[evz199-B48] Miglietta MP, McNally L, Cunningham CW. 2010 Evolution of calcium-carbonate skeletons in the Hydractiniidae. Integr Comp Biol. 50(3):428–435. 21558213 10.1093/icb/icq102

[evz199-B49] Morrow C, Cárdenas P. 2015 Proposal for a revised classification of the Demospongiae (Porifera). Front Zool. 12:7. 25901176 10.1186/s12983-015-0099-8 PMC4404696

[evz199-B50] Moya A, et al 2008 Carbonic anhydrase in the scleractinian coral *Stylophora pistillata*: characterization, localization, and role in biomineralization. J Biol Chem. 283(37):25475–25484. 18617510 10.1074/jbc.M804726200

[evz199-B51] Naggi A, et al 2018 Structure and function of stony coral intraskeletal polysaccharides. ACS Omega 3(3):2895–2901. 30221225 10.1021/acsomega.7b02053 PMC6130787

[evz199-B52] Petersen TN, Brunak S, von Heijne G, Nielsen H. 2011 SignalP 4.0: discriminating signal peptides from transmembrane regions. Nat Methods. 8(10):785–786. 21959131 10.1038/nmeth.1701

[evz199-B53] Puverel S, Tambutté E, Pereira-Mouriès L et al 2005 Soluble organic matrix of two Scleractinian corals: partial and comparative analysis. Comp Biochem Physiol B Biochem Mol Biol. 141(4):480–487. 15982916 10.1016/j.cbpc.2005.05.013

[evz199-B54] Puverel S, Tambutté E, Zoccola D, et al 2005 Antibodies against the organic matrix in scleractinians: a new tool to study coral biomineralization. Coral Reefs. 24(1):149–156.

[evz199-B55] Rahman MA, Isa Y, Uehara T. 2006 Studies on two closely related species of octocorallians: biochemical and molecular characteristics of the organic matrices of endoskeletal sclerites. Mar Biotechnol. 8(4):415–424. 16670968 10.1007/s10126-005-6150-6

[evz199-B56] Ramos-Silva P, et al 2013 The skeletal proteome of the coral *Acropora millepora*: the evolution of calcification by co-option and domain shuffling. Mol Biol Evol. 30(9):2099–2112. 23765379 10.1093/molbev/mst109 PMC3748352

[evz199-B57] Reggi M, et al 2016 Influence of intra-skeletal coral lipids on calcium carbonate precipitation. CrystEngComm. 18(46):8829–8833.

[evz199-B58] Reyes-Bermudez A, Lin Z, Hayward DC, Miller DJ, Ball EE. 2009 Differential expression of three galaxin-related genes during settlement and metamorphosis in the scleractinian coral *Acropora millepora*. BMC Evol Biol. 9:178. 19638240 10.1186/1471-2148-9-178 PMC2726143

[evz199-B59] Riesgo A, Farrar N, Windsor PJ, Giribet G, Leys SP. 2014 The analysis of eight transcriptomes from all poriferan classes reveals surprising genetic complexity in sponges. Mol Biol Evol. 31(5):1102–1120. 24497032 10.1093/molbev/msu057

[evz199-B60] Romano SL, Cairns SD. 2000 Molecular phylogenetic hypotheses for the evolution of Scleractinian corals. Bull Mar Sci. 67:1043–1068.

[evz199-B61] Romano SL, Palumbi SR. 1996 Evolution of Scleractinian corals inferred from molecular systematics. Science 271(5249):640–642.

[evz199-B62] Ronquist F, et al 2012 MrBayes 3.2: efficient Bayesian phylogenetic inference and model choice across a large model space. Syst Biol. 61(3):539–542. 22357727 10.1093/sysbio/sys029 PMC3329765

[evz199-B63] Sanchez S, Hourdez S, Lallier FH. 2007 Identification of proteins involved in the functioning of *Riftia pachyptila* symbiosis by subtractive suppression hybridization. BMC Genomics 8:337. 17892591 10.1186/1471-2164-8-337 PMC2175520

[evz199-B64] Sevilgen DS, et al 2019 Full in vivo characterization of carbonate chemistry at the site of calcification in corals. Sci Adv. 5(1):eaau7447. 30746460 10.1126/sciadv.aau7447 PMC6357752

[evz199-B65] Shinzato C, et al 2011 Using the *Acropora digitifera* genome to understand coral responses to environmental change. Nature 476(7360):320–323. 21785439 10.1038/nature10249

[evz199-B66] Simão FA, Waterhouse RM, Ioannidis P, Kriventseva EV, Zdobnov EM. 2015 BUSCO: assessing genome assembly and annotation completeness with single-copy orthologs. Bioinformatics 31(19):3210–3212. 26059717 10.1093/bioinformatics/btv351

[evz199-B67] Simion P, et al 2017 A large and consistent phylogenomic dataset supports sponges as the sister group to all other animals. Curr Biol. 27(7):958–967. 28318975 10.1016/j.cub.2017.02.031

[evz199-B68] Sodergren E, Weinstock GM, Davidson EH. 2006. The genome of the sea urchin Strongylocentrotus purpuratus. Science 314:941–952. 10.1126/science.1133609 PMC3159423 17095691

[evz199-B69] Sonnhammer EL, von Heijne G, Krogh A. 1998 A hidden Markov model for predicting transmembrane helices in protein sequences. Proc Int Conf Intell Syst Mol Biol. 6:175–182. 9783223

[evz199-B70] Srivastava M, et al 2010 The *Amphimedon queenslandica* genome and the evolution of animal complexity. Nature 466(7307):720. 20686567 10.1038/nature09201 PMC3130542

[evz199-B71] Stolarski J, et al 2011 The ancient evolutionary origins of Scleractinia revealed by azooxanthellate corals. BMC Evol Biol. 11:316. 22034946 10.1186/1471-2148-11-316 PMC3224782

[evz199-B72] Sunagawa S, DeSalvo MK, Voolstra CR, Reyes-Bermudez A, Medina M. 2009 Identification and gene expression analysis of a taxonomically restricted cysteine-rich protein family in reef-building corals. PLoS One 4(3):e4865. 19283069 10.1371/journal.pone.0004865 PMC2652719

[evz199-B73] Takeuchi T, Yamada L, Shinzato C, Sawada H, Satoh N. 2016 Stepwise evolution of coral biomineralization revealed with genome-wide proteomics and transcriptomics. PLoS One 11(6):e0156424. 27253604 10.1371/journal.pone.0156424 PMC4890752

[evz199-B74] Tambutté S, et al 2011 Coral biomineralization: from the gene to the environment. J Exp Mar Biol Ecol. 408(1–2):58–78.

[evz199-B75] Van IH, et al 2016 Origin and early diversification of phylum Cnidaria: key macrofossils from the Ediacaran system of North and South America In: The Cnidaria, past, present and future. Cham: Springer p. 31–40.

[evz199-B76] Voigt O, Adamski M, Sluzek K, Adamska M. 2014 Calcareous sponge genomes reveal complex evolution of α-carbonic anhydrases and two key biomineralization enzymes. BMC Evol Biol. 14:230. 25421146 10.1186/s12862-014-0230-z PMC4265532

[evz199-B77] Von Euw S, et al 2017 Biological control of aragonite formation in stony corals. Science 356(6341):933–938. 28572387 10.1126/science.aam6371

[evz199-B78] Wheeler AP, George JW, Evans CA. 1981 Control of calcium carbonate nucleation and crystal growth by soluble matrx of oyster shell. Science 212(4501):1397–1398. 17746262 10.1126/science.212.4501.1397

[evz199-B79] Wild C, et al 2011 Climate change impedes scleractinian corals as primary reef ecosystem engineers. Mar Freshwater Res. 62(2):205–215.

[evz199-B80] Wood R. 1999 Reef evolution. Oxford, UK: Oxford University Press on Demand.

[evz199-B81] Zoccola D, et al 2015 Bicarbonate transporters in corals point towards a key step in the evolution of cnidarian calcification. Sci Rep. 5:9983. 26040894 10.1038/srep09983 PMC4650655

