# Supplementary material for: New Non-Bilaterian Transcriptomes Provide Novel Insights into the Evolution of Coral Skeletomes
Source: Genome Biol Evol. 2019 Sep 13;11(11):3068–81. doi: 10.1093/gbe/evz199 (PMC6824150; doi:10.1093/gbe/evz199)
Supplement: evz199_Supplementary_Data [file evz199_supplementary_data.zip › Concietal_GBE_SMat_SFig_Legends.docx]

S.Mat1: Sequence ID and e-values for homologs of scleractinian and octocoral skeletogenic proteins identified using BLAST (BLASTp, cut-off < 1e^-09^).

S.Mat2: Taxonomy, accession numbers, and references for the sequence data utilized to construct the homolog database.

S.Mat3: Accession and sequence features (annotation, length, source organism, completeness) for the protein queries used.

S.Mat4: Scleractinian best-hits for non-scleractinian homologs of acidic proteins (BLASTp, cut-off < 1e^-09^).

S. Fig1: Alignment of USOMP5-like sequences showing the position of the homologous superfamily Fibrinogen, alpha/beta/gamma-chain, C-terminal globular, subdomain 1 (IPR014716) in homoscleromorph sponges (red boxes) and *Aiptasia* (blue boxes). No domain was detected in *A. millepora.*

S. Fig2: Maximum Likelihood tree (500 bootstrap replicates) of cnidarian acidic proteins. Protein sequences aligned with MUSCLE. Best-fit model: WAG + Γ + I. Black dot on node indicates full support (100 bootstrap - 1.0 Posterior Probability). Bootstrap values in bold: support is >50 also in phylogeny based on MAFFT alignment.

S. Fig3: Phylogenetic analysis (500 bootstraps) of metazoan galaxin-related proteins. Tree displayed in figure based on protein sequences aligned with MUSCLE alignment. Bold number: node supported (>50) also in MAFFT phylogeny. Dot on node indicates full support (100 bootstrap - 1.0 Posterior Probability) in both phylogenies. Support for nodes <50 not shown regardless of posterior probability value.

S. Fig4: MAFFT-based phylogenetic analysis of galaxin-related proteins highlighting the presence of the RXRR motif described in Fukuda et al. (2003).

S. Fig5: Phylogenetic analysis (500 bootstrap replicates) of galaxin *sensu stricto* based on MUSCLE (a) and MAFFT (b) aligned sequences. Best-fit model for both alignments: JTT+Γ+I. Black dots on node indicates full support (100 bootstrap - 1.0 Posterior Probability). Maximum-likelihood and bayesian analyses were performed with PhyML 3.1 (in Seaview 4) and MrBayes 3.2.6, respectively. For the latter, a burn-in fraction of 20% was applied.

S. Fig6: Phylogenetic analysis (500 bootstrap replicates) of Collagen IV NC1 domain and putative homolog in spongins. Colored sequences (with exception of Q7JMZ8) were identified with BLASTp as part of this study. Other sequences from Aouacheria et al. (2006). Sequences were aligned in MUSCLE. Best-fit model: WAG+G+I. Numbers in bold: node support is >50 also in MAFFT-based phylogeny. Support showed for nodes of interest only.

S. Fig7: Phylogenetic analysis (500 bootstrap replicates) of Collagen IV NC1 domain and putative homolog in spongins. Colored sequences (with exception of Q7JMZ8) were identified with BLASTp as part of this study. Other sequences from Aouacheria et al. (2006). Sequences were aligned in MAFFT. Best-fit model: LG+G+I. Numbers in bold: node support is >50 also in MUSCLE-based phylogeny. Support showed for nodes of interest only.

S. Fig8: Phylogenetic analysis (500 bootstrap replicates) of octocoral carbonic anhydrases (CA) (MAFFT). Octocoral CAs are in blue. Sequences added to the dataset used in Lin et al. (2017). Best-fit model: LG+G. Black dots on node indicates full support (100 bootstrap - 1.0 Posterior Probability).

S. Fig9: Phylogenetic analysis (500 bootstrap replicates) of octocoral carbonic anhydrases (CA) (MUSCLE). Octocoral CAs are in blue. Sequences added to the dataset used in Lin et al. (2017). Best-fit model: LG+G. Black dots on node indicates full support (100 bootstrap - 1.0 Posterior Probability).

S. Fig10: Principal Component Analysis of acidic proteins. Aminoacid percentages within sequences and isoelectric point determined with ProtParam. Parameters calculated for complete sequences only. pI = isoelectric point, Lys = Lysine, Arg = Arginine, Glu = Glutamic acid, Asp = Aspartic acid, Ac.Bs_Ratio = Acidic-Basic ratio [(Asp+Glu)/(Arg+Lys)].

S. Fig11: Distribution of aspartic acid content within anthozoan proteins. Numbers in a white box: average content (%) ± standard deviation. Data estimated from protein sequences predicted from genomic data only. Data and scripts used to produce the graph available in the project repository.

S. Fig12: Distribution of lysine content within anthozoan proteins. Numbers in a white box: average content (%) ± standard deviation. Data estimated from protein sequences predicted from genomic data only. Data and scripts used to produce the graph available in the project repository.
